# Hospital-onset bacteraemia and fungaemia as a novel automated surveillance indicator: results from four European university hospitals, 2018 to 2022

**DOI:** 10.2807/1560-7917.ES.2025.30.24.2400613

**Published:** 2025-06-19

**Authors:** Seven JS Aghdassi, Suzanne D van der Werff, Gaud Catho, Manon Brekelmans, Luis A Peña Diaz, Niccolò Buetti, Ferenc D Rüther, Daniel Teixeira, Daniel Sjöholm, Pontus Nauclér, Michael Behnke, Maaike SM van Mourik, the PRAISE-HOB working group, PRAISE-HOB working group, Heike Düsseldorf, Anders F Johansson, Elisabeth Presterl, Louise Stougaard, Tjallie van der Kooi

**Affiliations:** 1 Charité – Universitätsmedizin Berlin, corporate member of Freie Universität Berlin and Humboldt-Universität zu Berlin, Institute of Hygiene and Environmental Medicine, Berlin, Germany; 2 Berlin Institute of Health at Charité – Universitätsmedizin Berlin, BIH Biomedical Innovation Academy, BIH Charité Digital Clinician Scientist Program, Berlin, Germany; 3 Department of Medicine Solna, Division of Infectious Diseases, Karolinska Institutet, Stockholm, Sweden; 4 Department of Infectious Diseases, Karolinska University Hospital, Stockholm, Sweden; 5 Infection Control Program and WHO Collaborating Centre, University of Geneva Hospitals and Faculty of Medicine, Geneva, Switzerland; 6 Infectious diseases division, Central Institute, Valais Hospital, Sion, Switzerland; 7 Department of Medical Microbiology and Infection Control, University Medical Centre Utrecht, Utrecht, the Netherlands; 8 Centre for Infectious Diseases Control, National Institute for Public Health and the Environment, Bilthoven, the Netherlands; 9 Université Paris Cité, Inserm, IAME, Paris, France; 10 Department of Medicine Solna, Division of Clinical Epidemiology, Karolinska Institutet, Stockholm, Sweden; 11 The additional members of the network are listed under Collaborators and at the end of the article

**Keywords:** hospital-onset, bacteraemia, fungaemia, surveillance, bloodstream infection, automated surveillance

## Abstract

**BACKGROUND:**

Conventional manual surveillance of healthcare-associated infections is labour-intensive and therefore often restricted to areas with high-risk patients. Fully automated surveillance of hospital-onset bacteraemia and fungaemia (HOB) may facilitate hospital-wide surveillance.

**AIM:**

To develop an algorithm and minimal dataset (MDS) required for automated surveillance of HOB and apply it to real-life routine data in four European hospitals

**METHODS:**

Through consensus discussion, a HOB definition with MDS suitable for automated surveillance was developed and applied in a retrospective multicentre observational study including all adult patients admitted to hospitals in the Netherlands, Germany, Sweden and Switzerland (2018–22). Annual HOB rates were calculated per 1,000 patient days for hospital, intensive care unit (ICU) and non-ICU settings.

**RESULTS:**

HOB was defined as a positive blood culture with a recognised pathogen 2 or more days after hospital admission. For common commensals, two blood cultures with the same commensal within 2 days were required. HOB rates were comparable between the four hospitals (1.0–2.2/1,000 patient days). HOB rates were substantially higher in ICU than non-ICU settings, and HOB with common commensals accounted for 14.8–28.2% of all HOB. HOB rates per 1,000 patient days were consistent over time, but higher in 2020–21. HOB caused by *Staphylococcus aureus* comprised 8.4–16.0% of all HOB.

**CONCLUSION:**

Automated HOB surveillance using a common definition was feasible and reproducible across four European hospitals. Future studies should investigate clinical relevance and preventability of HOB, and focus on strategies to make the automated HOB metric an actionable infection control tool.

**Key public health message**

**What did you want to address in this study?**

Infections can occur when receiving hospital care. Measuring these is an important step to reducing these infections. However, this is often only done in certain hospital areas and involves time-consuming manual checking of many patient charts. We introduce a fully automatic method that can be used throughout the hospital to measure infections caused by bacteria and fungi in the blood, so-called hospital-onset bacteraemia and fungaemia (HOB).

**What have we learnt from this study?**

Our new method was successfully used in four large hospitals in four different European countries between 2018 and 2022. The number of times HOB was reported was comparable between all four hospitals and closely followed the number of blood samples that were taken. We observed some differences in the most common bacteria and fungi causing HOB in each hospital.

**What are the implications of your findings for public health?**

We show that, with our method, it is possible to automatically measure HOB in hospitals. This HOB measure will be a useful first step when switching from traditional measuring by manual chart review to automatic digital measuring of infections. We provide a guide for other hospitals and researchers to set-up this automatic digital measuring of HOB.

## Introduction

Surveillance of healthcare-associated infections (HAI) is a cornerstone of infection prevention and control (IPC) and an effective means to reduce the incidence of HAI [1-3]. Current surveillance systems rely primarily on manual chart review to detect infections, which is resource-intensive, time-consuming and prone to subjective interpretation [4]. Automated surveillance (AS) systems utilise algorithms to complement or replace certain steps of manual surveillance by analysing routine care data that are automatically extracted from electronic health records (EHR). Automated surveillance systems offer the potential to obtain surveillance data in a timelier manner, to increase standardisation and interpretability of results, and once set-up, to be less time-consuming [5-7].

Currently, AS systems are mostly limited to research settings and single institutions and hence vary considerably in terms of methods [7]. As a result, there is a great need for HAI definitions and targets suitable for AS, reliable detection algorithms and data structures that promote interoperability of medical data across different institutions. The PRAISE (Providing a Roadmap for Automated Infection Surveillance in Europe) network aims to provide methods and resources for facilitating the transition of AS from the research setting to large-scale implementation [8,9]. Multi-institutional development of AS methods will be crucial to maintain comparability of surveillance outcomes, one of the essential aspects of surveillance.

Hospital-onset bacteraemia and fungaemia (HOB) has been suggested as a novel surveillance and quality indicator and has been considered a suitable primary target for large-scale AS implementation, given the relative simplicity of the case definition with a focus on microbiological results from blood cultures [10]. Compared to conventional manual surveillance of central line-associated bloodstream infections (CLABSI), automated HOB surveillance may enable coverage of the entire inpatient population, i.e. surveillance of all admitted patients not just selected populations, and an increased scope of the surveillance target, by also capturing bloodstream infections (BSI) not associated with intravascular catheters [11]. While the potential of HOB surveillance has been discussed in various publications over the past few years [11-13], a detailed HOB definition and method that is suitable for fully automated surveillance, has not been published to date.

In this article, we report the collaborative development of an automated HOB surveillance algorithm within the PRAISE network and its application to retrospective data from four European university hospitals. We aim to illustrate potential applications of HOB surveillance to guide quality improvement and identify questions for future research.

## Methods

### Development of PRAISE-consensus HOB definition

The HOB definition and minimal dataset (MDS) were developed through iterative discussion between members within the PRAISE network. The PRAISE network was formed in 2019 with the ambition to support the development and implementation of automated surveillance in Europe [8]. Since 2021, a dedicated HOB working group has focused on developing a HOB definition suitable for large-scale automated surveillance. This PRAISE-HOB working group includes infectious disease and IPC specialists, epidemiologists and software developers from eight European countries, representing hospitals as well as national reference centres for HAI surveillance.

The following general principles guided the development: feasibility of implementation in all hospitals or hospital networks involved, alignment with existing surveillance methods where possible, and achieving unambiguous definitions of HOB suitable for automated surveillance. No formal consensus methods were used during the development process. Differences were resolved through discussion and, in case of doubt, explorative sensitivity analyses were performed to assess the impact of decisions. In general, existing concepts from surveillance definitions were re-used where possible, to optimise consistency. For example, the hospital-onset period was set to start on day 2 of admission (with day of admission being day 0) as per National Healthcare Safety Network (NHSN) HAI classification of the United States (US) Centers for Disease Control and Prevention (CDC) [14].

### Study design and study population

The HOB algorithm was applied in a retrospective cohort study including all patients admitted to one of the hospitals participating in the project (with access to source data) during the study period (University Medical Centre Utrecht (Utrecht, the Netherlands), Charité-University Hospital (Berlin, Germany), Karolinska University Hospital (Stockholm, Sweden) and Geneva University Hospitals (Geneva, Switzerland)). University Medical Centre Utrecht has around 1,000 inpatient beds, 27,000 annual inpatient admissions and 170,000 annual patient days. Charité-University Hospital has approximately 3,000 inpatient beds, 120,000 annual inpatient admissions and 800,000 annual patient days. Karolinska University Hospital has around 1,100 inpatient beds, 70,000 inpatient admissions and 370,000 annual patient days. Geneva University Hospital has approximately 2,100 inpatient beds, 50,000 annual inpatient admissions and 700,000 annual patient days. In this paper, the hospitals were anonymised and will be referred to as Hospitals 1 to 4. All hospitals are publicly funded tertiary care hospitals, i.e. providing maximum care as per national classification.

The study period varied across hospitals ranging from 2018 to 2022. In this study, paediatric patients, defined as age < 18 or admissions to a paediatric ward, were excluded from the analysis. Moreover, certain wards providing mainly psychiatric, rehabilitation or short-term care were excluded at the discretion of each hospital. In these cases, denominator data and blood cultures collected from these wards were not included in the denominator or the algorithm. 

### Algorithm application

All hospitals applied the algorithm locally and only the aggregated outcomes were combined in this manuscript. Data were collected electronically from EHR systems (microbiology results, patient movement data, see Supplementary Material S1 pages 13–16 for details of the data specification). To assess whether all algorithms developed by the participating hospitals yielded comparable results, an in-person meeting was organised (July 2023), where only algorithm implementation scripts were exchanged and run on real-life EHR data as well as fictional test data including rare or extreme scenarios, i.e. no patient data were shared. These datasets were formatted according the MDS.

Algorithms were programmed in different programming languages, depending on the hospital’s expertise and local conventions (SAS enterprise guide, R version 4.1.0, JAVA, or webservice using C# and .Net Core v6).

### Analysis

This manuscript includes an overall presentation of HOB epidemiology in the four European hospitals and illustrates possible applications of HOB surveillance. We present yearly HOB rates, differentiated by pathogen or common commensal, intensive care unit (ICU) or non-ICU, as well as trends over time. Rates are calculated per 1,000 inpatient days. We do not aim to compare rates directly. Instead, we present first insights into the epidemiology of this standardised metric. Furthermore, the frequency of sampling of blood cultures taken on day 2 after hospital admission or later was depicted to explore the relationship of blood culture frequency with HOB rates. The usability of HOB as a surveillance metric will be further explored by examining the causative microorganisms and drawing careful conclusions from the pathogen distribution. For data presentation, microorganisms were separated into the following groups: anaerobes, coagulase-negative staphylococci, Enterobacterales, enterococci, other Gram-negative rods, *Pseudomonas* species, *Staphylococcus aureus*, streptococci, yeasts and other/unspecified.

## Results

### PRAISE-consensus HOB definition

**Figure 1:**
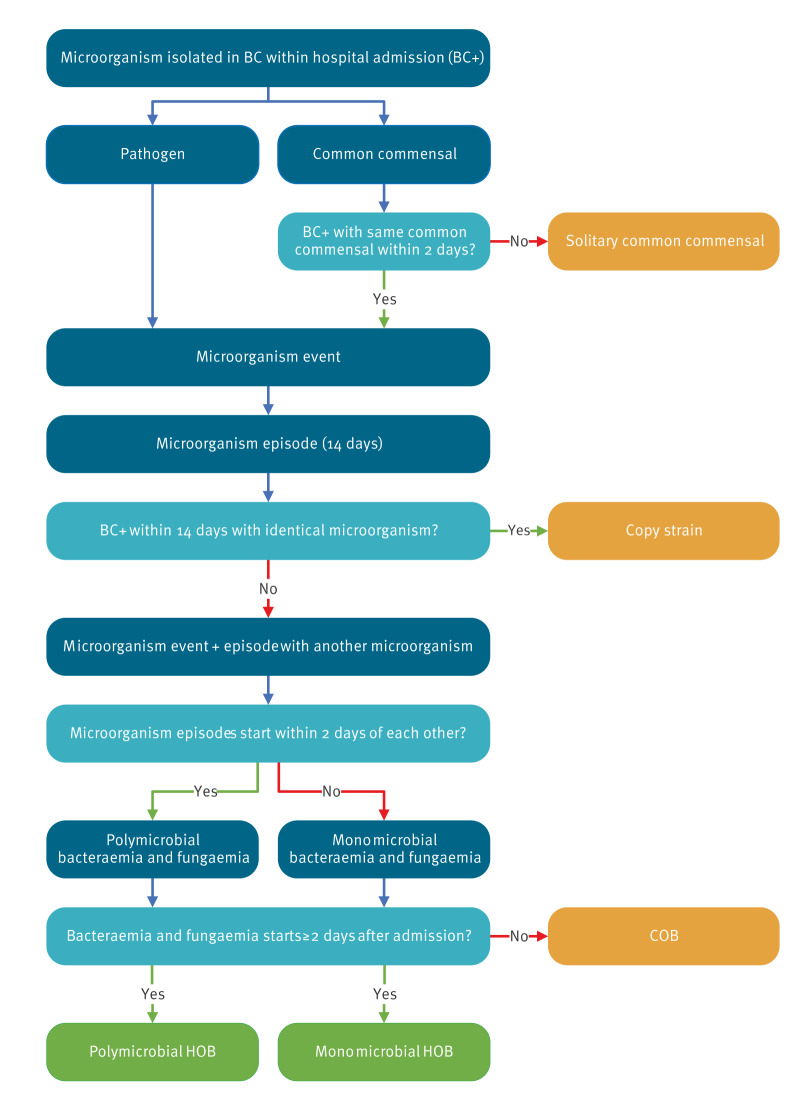
Schematic representation of the HOB algorithm

BC: blood culture; BC+: positive blood culture; COB: community-onset bacteraemia and fungaemia: HOB: hospital-onset bacteraemia and fungaemia.

The final consensus algorithm is presented and summarised in Figure 1.

This is a simplified version of the HOB algorithm that is not intended for real-time processing of data, but to visualise how the different concepts relate to each other. For more details on the HOB consensus definition, including detailed flowchart and examples, please refer to Supplementary Material S1 pages 4-12.

In short, microorganisms identified in blood cultures obtained during hospital admissions are classified as either a pathogen or common commensal as per NHSN CDC classification [15]. A positive blood culture with a pathogen or two repeated blood cultures with common commensals within 2 days constitute a ‘microorganism event’. Solitary common commensals, i.e. common commensals without a confirmation within 2 days, are disregarded. A microorganism event marks the onset of a ‘microorganism episode’ with a duration of 14 days or until discharge, whichever comes first. During a microorganism episode, the same microorganism in other blood cultures is considered a repeated isolate (copy strain) and does not constitute a new episode. Microorganism episodes starting within 2 days of each other are grouped into ‘polymicrobial episodes’ as they likely represent one clinical event. Finally, the microorganism episodes are classified as hospital-onset, i.e. HOB, if they start on day 2 of admission or later (with day 0 being the day of admission). Of note, multiple (overlapping) HOB episodes are possible, for example when different microorganisms are recovered from blood cultures drawn later in time. HOB episodes are attributed to the ward where the patient was 2 days prior to the start of the episode. Patient days are counted by ward and by groups of wards, e.g. ward type or specialty. In this manuscript, all mentions of bacteraemia reflect bacteraemia and fungaemia.

More details of the consensus definition, including examples, a more in-depth depiction of the algorithm, the MDS specification, and details of denominator data calculation are described in Supplementary Material S1. During the in-person testing of the algorithm, all local algorithms detected identical HOB episodes numbers in all four real life datasets and the test data.

### Cohort description

The four hospitals provided data for variable periods between 2018 and 2022. During the observation period, 2,100 bacteraemia episodes were observed in Hospital 1, of which 996 (47.4%) were HOB. In Hospital 2, 15,096 bacteraemia episodes were recorded, of which 6,895 (45.7%) were HOB. In Hospital 3, 5,411 bacteraemia episodes were recorded, of which 1,697 (31.4%) were HOB. In Hospital 4, there were 6,964 bacteraemia episodes, of which 2,499 (35.8%) were HOB. Table 1 illustrates the analysed dataset by hospital. Of note, the balance between pre-COVID-19 and COVID-19 pandemic years differed by hospital, possibly impacting the aggregated HOB rates.

**Table 1:** Description of dataset and rates of HOB per hospital for the entire observation period, four European hospitals, 2018–2022

Parameter	Hospital 1	Hospital 2	Hospital 3	Hospital 4
Observation period	2018–21	2018–22	2018–20	2018–22
Patient days	454,045	3,208,694	881,038	2,406,577
Blood cultures taken	24,392	226,442	47,501	116,566
Blood cultures per 1,000 patient days	53.7	70.6	53.9	48.4
Blood culture positivity rate (%)	12.1	8.9	10.2	6.8
HOB episodes	996	6,895	1,697	2,499
HOB episodes per 1,000 patient days	2.2	2.1	1.9	1.0
HOB episodes with CC (% of all HOB)	281 (28.2)	1,363 (19.8)	364 (21.4)	370 (14.8)
HOB episodes with CC per 1,000 patient days	0.6	0.4	0.4	0.2
HOB episodes with pathogen (% of all HOB)	785 (78.8)	5,715 (82.9)	1,405 (82.8)	2,173 (87.0)
HOB episodes with pathogen per 1,000 patient days	1.7	1.8	1.6	0.9
Polymicrobial HOB episodes (% of all HOB)	159 (16.0)	849 (12.3)	226 (13.3)	303 (12.1)
Polymicrobial HOB episodes per 1,000 patient days	0.4	0.3	0.3	0.1
Median in-hospital days until onset of HOB (IQR)^a^	12 (6–23)	14 (7–29)	9 (4–18)	28 (8–32)

CC: common commensal; HOB: hospital-onset bacteraemia and fungaemia; IQR: interquartile range.

^a^ Hospital 4, unlike the other three hospitals, consists of several hospitals organised in a network treated as one hospital. Therefore, patients are not considered as readmitted when transferred between different hospitals, resulting in longer median and IQR admission time.

Data depicted pertains only to wards included in the study. Multiple episodes with different microorganisms occurring within a 2-day period were counted as one polymicrobial HOB. Since polymicrobial HOB with both pathogen and common commensal were possible, the sum of HOB with pathogen and HOB with common commensal is greater than or equal to the number of all HOB. For counting blood cultures, only those taken from day two onwards were considered to align with HOB counting. Data represent (n) unless otherwise specified.

The number of blood cultures per 1,000 patient days ranged between 48.4 (Hospital 4) to 70.6 (Hospital 2). HOB rates per 1,000 patient days were comparable between the four hospitals and ranged from 1.0 to 2.2. The share of HOB with common commensals and pathogens as well as polymicrobial HOB among all HOB was comparable across hospitals. In all four hospitals, the median duration between hospital admission and onset of HOB was over 1 week, with a range of 9 (Hospital 3) to 28 (Hospital 4) days. Hospital 4 has a longer median and IQR admission time than the other three hospitals as it actually is several hospitals organised in a network treated as one hospital. Therefore, patients are not considered as readmitted when transferred between different hospitals. Of all those admitted with a HOB, 9.4–28.2% patients experienced at least one additional HOB during their admission, and between 4.3 and 7.5% of patients experienced a partial temporal overlap of at least two HOB episodes.

Table 2 displays the analysed dataset per hospital separately for ICU and non-ICU. In all four hospitals, blood culture sampling frequency and HOB rates per 1,000 patient days were higher in ICU than non-ICU. The proportion of positive blood cultures among all blood cultures taken, however, showed only small differences between ICU and non-ICU. Similarly, the shares of HOB with common commensals and pathogens were comparable between ICU and non-ICU when considered for the individual hospitals.

**Table 2:** Description of dataset and rates of HOB in intensive care units (ICU) and non-ICU per hospital for the entire observation period, four European hospitals, 2018–2022

Parameter	Hospital 1		Hospital 2		Hospital 3		Hospital 4	
	ICU	Non-ICU	ICU	Non-ICU	ICU	Non-ICU	ICU	Non-ICU
Observation period	2018–21		2018–22		2018–20		2018–22	
Patient days	33,335	420,710	448,148	2,760,546	43,581	837,457	50,715	2,355,862
Blood cultures taken	8,175	16,217	120,023	106,419	12,913	34,588	12172	104,394
Blood cultures per 1,000 patient days	245.2	38.5	267.8	38.5	296.3	41.3	242.6	44.3
Blood culture positivity rate (%)	14.0	11.1	8.4	9.5	8.1	11.0	6.5	6.9
HOB episodes	298	698	2,689	4,206	304	1,393	165	2,334
HOB episodes per 1,000 patient days	8.9	1.7	6.0	1.5	7.0	1.7	3.2	1.0
HOB episodes with CC (% of all HOB)	104 (34.9)	178 (25.5)	529 (19.7)	834 (19.8)	79 (26.0)	285 (20.5)	22 (13.3)	348 (14.9)
HOB episodes with CC per 1000 patient days	3.1	0.4	1.2	0.3	1.8	0.3	0.4	0.2
HOB episodes with pathogen (% of all HOB)	230 (77.2)	557 (79.8)	2,231 (83.0)	3,484 (82.8)	244 (80.3)	1,161 (83.3)	144 (87.3)	2,029 (86.9)
HOB episodes with pathogen per 1,000 patient days	6.9	1.3	5.0	1.3	5.6	1.4	2.8	0.9
Polymicrobial HOB episodes (% of all HOB)	54 (18.1)	105 (15.0)	316 (11.8)	533 (12.7)	40 (13.2)	186 (13.4)	15 (9.1)	288 (12.3)
Polymicrobial HOB episodes per 1,000 patient days	1.6	0.2	0.7	0.2	0.9	0.2	0.3	0.1
Median in-hospital days until onset of HOB (IQR)^a^	12 (7–22)	12 (6–23)	19 (9–38)	12 (5–23)	12 (6–21)	9 (4–17)	21 (7–22)	29 (7–33)

CC: common commensal; HOB: hospital-onset bacteraemia and fungaemia; ICU: intensive care unit; IQR: interquartile range.

^a^ Hospital 4, unlike the other three hospitals, consists of several hospitals organised in a network treated as one hospital. Therefore, patients are not considered as readmitted when transferred between different hospitals, resulting in longer median and IQR admission time.

Data depicted in this table pertain only to wards included in the study. Multiple HOB with different microorganisms occurring within a two-day period were counted as one polymicrobial HOB. Since polymicrobial HOB with both pathogen and common commensal were possible, the sum of HOB with pathogen and HOB with common commensal is greater than or equal to the number of all HOB. For counting blood cultures, only those taken from day two onwards were considered to align with HOB counting. Data represent (n) unless otherwise specified.

### Trends

**Figure 2:**
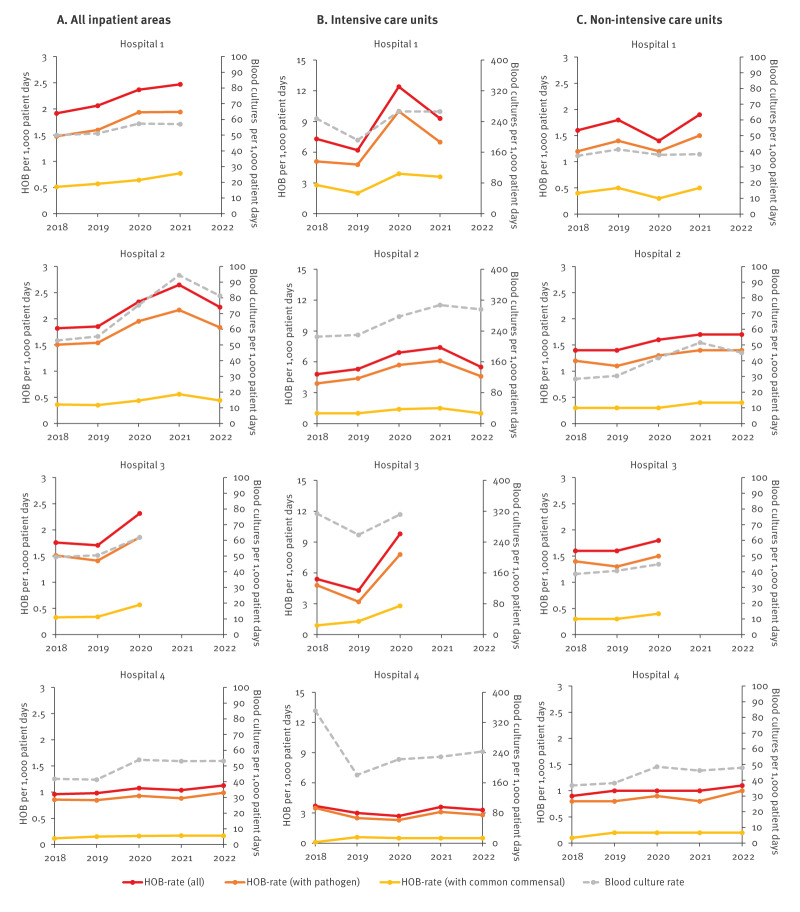
Rates of HOB and blood culture frequency over time per hospital, four European hospitals, 2018–2022

HOB: hospital-onset bacteraemia and fungaemia; ICU: intensive care unit.

Figure 2A shows HOB rates and the number of blood cultures per 1,000 patient days over time for all included inpatient areas separately for the four hospitals, while Figure 2B and Figure 2C pertain exclusively to data from ICU and non-ICU, respectively. In all hospitals, a correlation between HOB rates and blood culture frequency was observed, which remained stable over time. This was the case for all HOB combined, and for HOB with common commensals and HOB with pathogens considered separately. However, when looking at ICU only, HOB rates and blood culture frequency were higher during the years 2020 and 2021, coinciding with the COVID-19 pandemic.

Panel A pertains to all inpatient areas of the hospital, B pertains only to intensive care units, and C pertains only to non-intensive care units.

### Causative microorganisms

**Figure 3:**
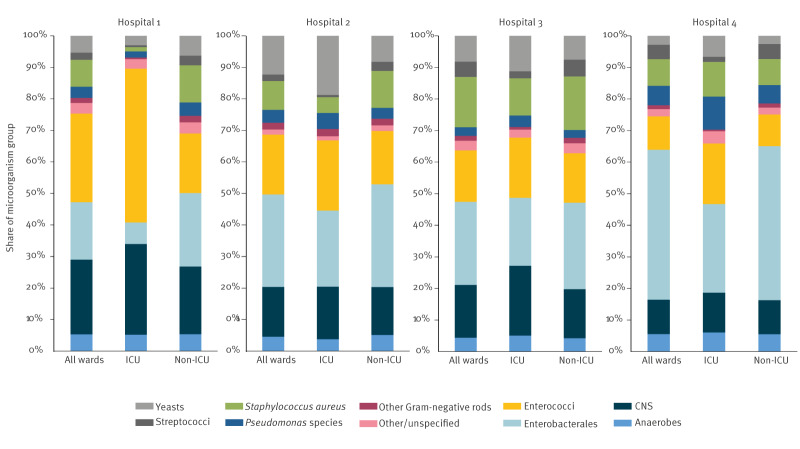
Share of microorganism group among all microorganisms in HOB episodes per hospital, four European hospitals, 2018–2022

CNS: coagulase-negative staphylococci; HOB: hospital-onset bacteraemia and fungaemia; ICU: intensive care unit.

Microorganisms in HOB showed variability between the four hospitals, with enterococci accounting for a larger portion of microorganisms in HOB in Hospital 1 than in the other hospitals and for example more Enterobacterales in Hospital 4. Moreover, differences between ICU and non-ICU were observed, with a general trend of more enterococci HOB in ICU across all four hospitals. Conversely, the proportion of HOB caused by Enterobacterales was higher in non-ICU than ICU. Of note, HOB caused by *Staphylococcus aureus* accounted for approximately 8.6% of HOB in Hospital 1, 9.1% in Hospital 2, 16.0% in Hospital 3, and 8.4% in Hospital 4. More details are depicted in Figure 3.

## Discussion

This multicentre retrospective cohort study is, to our knowledge, the first published fully automated and standardised multinational application of an operational definition of HOB for surveillance that could be implemented and used in practice in the near future. We demonstrate the feasibility of developing and applying a consensus definition for the fully automated surveillance of HOB in four European hospitals and provide detailed guidance for future implementation. The incidence of HOB found in our study adds to results from other reports, ranging from 6.9 to 22.1 HOB per 1,000 patient days (3.2–8.9 in this study) in the adult ICU setting and around 1.8 per 1,000 patient days in the non-ICU setting (1.0–1.7 in this study) [10,16]. However, in many previous studies, important details of the definition are not always provided or differ across studies, precluding direct comparison of rates and epidemiological characteristics [10,13,17,18]. In the PRAISE-HOB definition, we have addressed these possible sources of inconsistency by providing detailed implementation guidance (provided in Supplementary Material S1).

The PRAISE-HOB consensus definition differs from recently published approaches, mainly in that positive blood cultures undergo classification prior to being counted as a HOB event, thereby aiming to improve the relevance of HOBs being counted. For example, single common commensals are never classified as HOB but discarded as solitary commensals, and the definition also specifies explicitly how to handle pre-existing community-onset bacteraemia. A further important element of the PRAISE-HOB definition that adds to previously published approaches is the systematic grouping of microorganism episodes starting within 2 days of each other as single bacteraemia episodes (polymicrobial HOB), as this most likely represents a single clinical event. Interestingly, the contribution of polymicrobial HOB is sizeable, representing 12 to 16% of HOB episodes in this study. Finally, we have set the episode duration, i.e. the timeframe during which onset of a new HOB with the same microorganisms is not possible, to 14 days but other specifications, e.g. 30 days can be found in the literature [19].

Application of the consensus definition to real-life data revealed both commonalities and differences in the epidemiology of HOB between four university hospitals in Europe. The patient population under consideration likely differed substantially across the four settings, hence the analysis does not aim to compare rates across hospitals. Strikingly however, the HOB rates were fairly consistent across the different hospitals and also the proportion of all bacteraemia episodes that were hospital-onset was comparable across the four hospitals. HOB surveillance may provide multiple insights to identify potential areas of quality improvement. For example, HOB surveillance allows for large-scale and consistent monitoring of the full breadth of the inpatient population. Interestingly, when looking at absolute numbers, the majority of HOB episodes in our study were attributable to non-ICU wards, likely resulting from the substantially higher number of non-ICU patient days. Nevertheless, the fact that HOB episodes are not only occurring in the ICU, and that in total numbers HOB are more frequent outside of the ICU, is likely not exclusive to our study [20]. This highlights the broad applicability of HOB surveillance to patients without central lines and of diverse underlying diseases, which represents a key advantage over CLABSI surveillance. HOB surveillance may also be employed to detect increased incidence of BSI over time. In our analysis, we observed an increase in HOB during the years most impacted by the COVID-19 pandemic (2020 and 2021), which is in alignment with other studies that have reported increased nosocomial BSI during the pandemic [21,22]. In addition, closer inspection of the local microorganism distribution can provide clues for targeted prevention efforts. For example, in Hospitals 1 and 4 respectively, the relatively large share of enterococci or Gram-negative HOB may lead to further investigations and pathogen-specific HOB rates, e.g. *S. aureus or Candida* sp. may also provide information that can inform IPC responses.

In this study, we did not perform an assessment of the sources of HOB. Recent studies have found that major sources include endovascular infections, gastrointestinal and abdominal infections, urinary tract infections, skin and soft tissue or surgical site infections, while in a considerable proportion, no known source could be detected [17]. Interestingly, although there seems to be correlation between HOB and CLABSI rates, their overlap appears to be only modest. In a recent study from India, only 13% of HOB represented CLABSI episodes [16]. Conversely, HOB rates showed a high correlation with CLABSI rates in a study focused on the US ICU setting; this difference may potentially be explained by the central line utilisation in ICUs [10]. To gain deeper insights into both source of HOB and its concordance with established surveillance metrics such as CLABSI, further research will be necessary, particularly with a focus on the epidemiology in Europe. Another aspect warranting further study is the preventability of HOB. Several studies have described and used methods to assess the preventability of HOB [23] and initial results demonstrate that over 50% of HOB might be potentially preventable [16,17,24]. Application of uniform definitions and methods across hospitals and countries, as demonstrated in this study, will likely be of great value when assessing preventability and characterising the clinical significance of HOB episodes. Similarly, future research is needed to determine the most suitable approach for reporting HOB-related results to stimulate improvements in the quality of care, and suitable means of benchmarking HOB data, considering extraneous factors affecting HOB rates, such as blood culture frequency or baseline risk.

The detailed consensus definition and detailed implementation guidance provided in this study allows for implementation in settings with different IT infrastructure. We expect it to also be feasible outside of the university hospital setting, as the data needed for implementation are likely available in most European EHR systems. Importantly however, giving priority to developing fully automated surveillance definition that is feasible in variable settings, meant that we purposefully excluded clinical details, possibly at the cost of accuracy in some cases. Given the intended broad applicability of the PRAISE HOB consensus definition, we believe it will serve as an important reference in the context of transitioning from manual to automated surveillance in Europe, and that it will be complementary to other activities on the matter at the national and international level in Europe [25,26].

This study has several limitations. The specifications of the HOB definitions were defined based on consensus discussion, supported by existing literature and sometimes by exploratory data analysis. The impact and suitability of certain choices, e.g. duration of episode and definition for polymicrobial HOB, on the outcomes will have to be investigated in follow-up studies. In addition, the retrospective data analysis was limited to existing patient cohorts, thereby resulting in time periods that were not fully overlapping between the four hospitals. Finally, at this stage of development, we did not include information on antimicrobial susceptibility into the algorithm and the data presented excludes the paediatric population. Moreover, as manual HOB surveillance was not performed in our centres using a similar definition, we did not validate the automated HOB algorithm against manual surveillance and we also did not validate our results directly against data show in EHR systems.

## Conclusion

The definition and method for automated HOB surveillance was successfully applied and reproduceable in four hospitals from four different European countries. This new consensus definition addresses the difficulty to transpose the existing definitions for surveillance to automated surveillance and provides an important basis for future development. Future studies will need to further characterise the conditions underlying HOB episodes, assess preventability and effective interventions, if and how this metric can be used for benchmarking and assessing hospital performance, and define the metric’s role in infection prevention programs.

## PRAISE-HOB working group

Authors and Heike Düsseldorf (https://orcid.org/0009-0004-6048-063X) - Department Hospital Epidemiology and Infection Control Medical University of Vienna, Vienna, Austria. Anders F Johansson (https://orcid.org/0000-0003-0548-5943) - Department of Clinical Microbiology, Umeå University, Umeå, Sweden. Elisabeth Presterl (https://orcid.org/0000-0003-4395-7403) - Department Hospital Epidemiology and Infection Control Medical University of Vienna, Vienna, Austria. Louise Stougaard - Department of Data Integration and Analysis, Statens Serum Institut, Copenhagen, Denmark. Tjallie van der Kooi (https://orcid.org/0000-0002-5346-7224) - National Institute for Public Health and the Environment, Centre for Infectious Disease Control, Bilthoven, the Netherlands.

## Supplementary Data

1436000 Supplement
